# Single Wall Carbon Nanotubes Based Cryogenic Temperature Sensor Platforms

**DOI:** 10.3390/s17092071

**Published:** 2017-09-10

**Authors:** Bogdan Florian Monea, Eusebiu Ilarian Ionete, Stefan Ionut Spiridon, Aurel Leca, Anda Stanciu, Emil Petre, Ashok Vaseashta

**Affiliations:** 1 Cryogenic pilot plant, National R&D Institute for Cryogenics and Isotopic Technologies—ICSI Rm. Valcea, Uzinei Street, No. 4, 250050 Rm. Valcea, Romania; bogdan.monea@icsi.ro (B.F.M.); ionut.spiridon@icsi.ro (S.I.S.); 2 Faculty of Automation, Computers and Electronics, University of Craiova, 107 Decebal Blvd., 200440 Craiova, Romania; Emil.Petre@automation.ucv.ro; 3 Laboratory of Magnetism and Superconductivity, National Institute of Materials Physics, Atomistilor Str., No. 405A, 077125 Magurele, Romania; aurel.leca@infim.ro (A.L.); anda.stanciu@infim.ro (A.S.); 4 International Clean Water Institute, VA and NJCU—A State University of New Jersey, Jersey City, NJ 07305, USA; prof.vaseashta@ieee.org

**Keywords:** single wall carbon nanotubes, cryogenic microsensor, very low temperature measurement, electrophoretic alignment, nanoscience

## Abstract

We present an investigation consisting of single walled carbon nanotubes (SWCNTs) based cryogenic temperature sensors, capable of measuring temperatures in the range of 2–77 K. Carbon nanotubes (CNTs) due to their extremely small size, superior thermal and electrical properties have suggested that it is possible to create devices that will meet necessary requirements for miniaturization and better performance, by comparison to temperature sensors currently available on the market. Starting from SWCNTs, as starting material, a resistive structure was designed. Employing dropcast method, the carbon nanotubes were deposited over pairs of gold electrodes and in between the structure electrodes from a solution. The procedure was followed by an alignment process between the electrodes using a dielectrophoretic method. Two sensor structures were tested in cryogenic field down to 2 K, and the resistance was measured using a standard four-point method. The measurement results suggest that, at temperatures below 20 K, the temperature coefficient of resistance average for sensor 1 is 1.473%/K and for sensor 2 is 0.365%/K. From the experimental data, it can be concluded that the dependence of electrical resistance versus temperature can be approximated by an exponential equation and, correspondingly, a set of coefficients are calculated. It is further concluded that the proposed approach described here offers several advantages, which can be employed in the fabrication of a microsensors for cryogenic applications.

## 1. Introduction

Temperature is one of the most important parameters to measure, especially when we refer to the cryogenic field. Cryogenics is the science of attaining very low temperatures and observing its effect on materials, being widely used in various domains such as aerospace, nuclear, medical, mechanical, chemical, and electrical technologies, or for studying nature. The units used for temperature measurements are Kelvin (K) or Celsius (°C). The relation between them is defined by the Internationally Temperature Scale of 1990 (ITS-90) as: K = °C + 273.16. ITS-90 defines the thermodynamic temperature scale from 0.65 K to the highest temperature measurable in terms of the Planck radiation law using monochromatic radiation [1]. It specifies several temperature ranges between 0.65 and 1357.77 K, and 17 fixed points, which are the equilibrium states of different pure substances [2]. Based on different temperature-dependent properties [3], various cryogenic temperature sensors have been developed. Most of the sensors used in research laboratories, pilot plants and large cryogenic facilities as temperature measurement equipment are resistors, transistors, diodes, thermocouples and capacitors. Several other temperature measurement techniques, such as vapor pressure, gas thermometer, acoustic, magnetic susceptibility and noise, require complicated measurement methodologies and devices, which severely constrain system design [4]. Furthermore, some of the semiconductors, such as Germanium and Carbon, have excellent thermometric properties at low temperatures [5]. In general, temperature sensors based on semiconducting material have negative temperature coefficient (NTC) resistance, where resistance increases with temperature decrease. 

Temperature sensors miniaturization is a challenging task, especially when the process needs accurate measurements at a specific location requiring fast response time, high sensitivity, and stability over time, interchangeability, low cost operation, measurement system simplicity and low power consumption, which further necessitates low heat dissipation rates [6]. 

Because of their physical dimensions and superior thermal, electrical and mechanical properties [7,8], carbon nanotubes (CNTs) are viable candidates to be used as sensing element in a cryogenic temperature microsensor. Since their discovery [9], CNTs have been extensively studied, especially in sensor design and for different types of measurement apparatus (e.g., chemical sensors) [10,11]. Depending on the diameter and chirality, CNTs are either metallic or present semiconductor behavior [12]. When used as sensors, CNTs can provide accurate measurements at nanoscale and reduce the possibility of interference due to the proximity effect [7], which is very important for the cryogenic applications, more so in complicated thermal flow system. In addition, the small size sensor implies very low power consumption, in the range of fractions of a milliwatt [13]. Notwithstanding abundant investigations on growth and fabrication of SWCNT between electrodes and its use as gas sensors, use of CNTs as temperature sensor has not been researched much thus far.

As an extension of research efforts towards development of cryogenic temperature sensors [6,14,15,16,17], taking advantage of the metallic or semiconducting nature of the purified SWCNTs [18], we studied a SWCNT based cryogenic temperature sensor, which can measure temperatures in a very low temperature domain, viz. 2–77 K. Therefore, we first studied the temperature dependence of CNT layers resistance between 2 K and 77 K. Subsequently, using the results thus obtained, we present the technique to form bonding bridges of single walled carbon nanotubes between two electrodes. We also describe our experimental findings on the resistance vs. temperature (R-T) characteristics of the proposed sensor structures. As research expands in this field, it is anticipated that the CNTs based sensors for temperature measurement, especially in the cryogenic field, will be intensely studied and new devices will be developed. The CNTs may emerge as the structure pillars of the future branches of nanoscience: nanoelectronics and nanodetection [14].

## 2. Materials and Methods—Sensors Preparation

In a similar manner as described in [14], commercially produced CNTs (single-walled, purchased from Sigma Aldrich, Munich, Germany, 60% purity) were mixed together with isopropyl alcohol (proportion 1/10). The resulted solution was rigorously mixed using a sonication bath with controlled inside temperature of 30 °C to avoid alcohol evaporation effect. After 60 min, the solution was considered as being completely homogenized and then was filtered and dried. 

**Figure 1:**
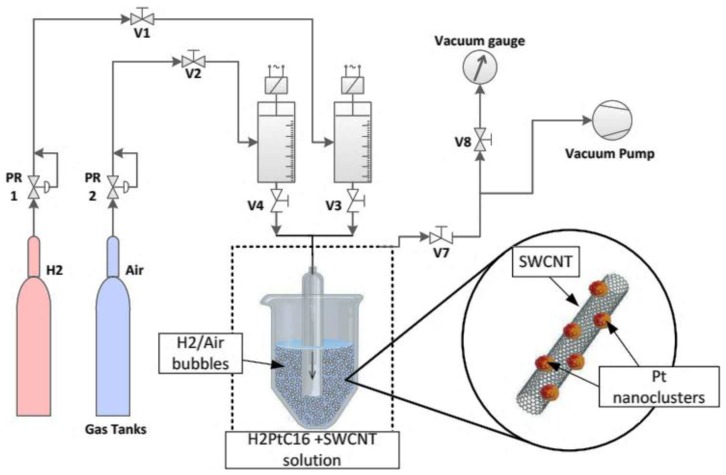
Schematic view of sensing fabrication unit with gas distribution system.

Before deposition of the platinum nanoclusters on the above-mentioned SWCNTs, the nanotubes suffered a functionalization and drying process. For the deposition process, a solution of H_2_PtCl_6_, 1% concentration, was obtained by dissolving chloroplatinic acid hexahydrate in distilled water. One milliliter of H_2_PtCl_6_ solution was extracted and introduced over SWCNTs together with high purity Hydrogen (5.0). A special bubbling device was used to limit the gas flow and the dimension of bubbles, as shown schematically in Figure 1.

For the following experiments, two sensor structures were obtained by employing a dropcast technique of a small quantity of solution (20 µL) between the electrodes. The first structure (here called Sensor 1) consists of two copper electrodes on a ceramic substrate with a distance of 1.25 mm between electrodes. The second structure (here called Sensor 2) consists of two gold electrodes on a Sitall substrate (crystalline glass-ceramic with ultra-low coefficient of thermal expansion) with a distance of 0.25 mm between electrodes. To align the SWCNTs and to form multilayers of nanotubes between the two electrodes [19,20], the structures that we prepared were subjected to a dielectrophoresis process by applying 1 VDC potential, for 1 min, over the electrodes. During this time, dry air at 60 °C was blown over the structures. Further, the sensors were subjected to a high temperature treatment (200 °C for 60 min) for achieving structure stability of the sensitive active layer.

## 3. Characterization—Results and Discussion

### 3.1. Characterization Methods

The two sensor structures prepared in this investigation were tested at very low temperature in a refrigeration unit Quantum Design Physical Property Measurement System (PPMS) with EverCool-II^®^ Cryogen-Free Cooling Technology, model P935A (referred as PPMS, Quantum Design, Inc., GmbH, Darmstadt, Germany). The PPMS provides a flexible automated workstation that can perform a variety of experiments requiring precise thermal control in the range 1.9 K to 400 K. The temperature measurement accuracy is ±0.5%, and temperature stability is ≤0.2% for temperatures ≤10 K and ≤0.02% for temperatures >10 K.

**Figure 2:**
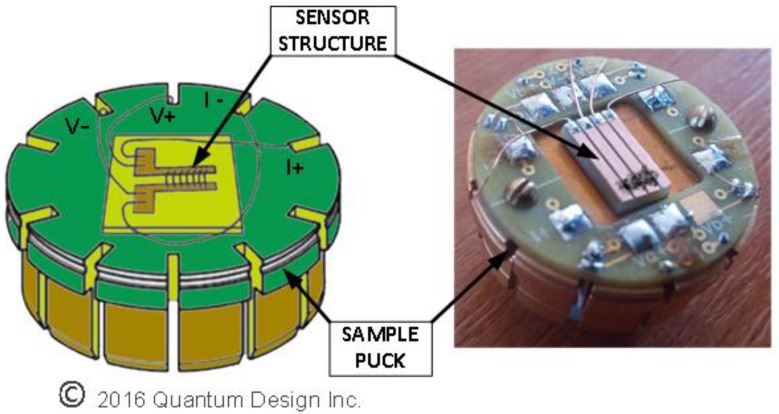
Device under test (DUT)—Four point connection to measure resistance.

To measure the resistance by the four-wire method, the structures (Sensor 1 and Sensor 2) were mounted on a standard PPMS sample puck (see Figure 2). Then, the sample puck was introduced in the PPMS sample chamber, the cold region of the refrigeration unit under investigation, which is constructed of copper to provide uniform temperature region. The sample puck is connected to the PPMS acquisition system using 12-pin connector placed on the bottom of the sample chamber. In a four-wire resistance measurement general configuration, the current is passed through a sample via two current leads, and two separate voltage leads measure the potential difference across the sample. To maintain a minimum value of the current adsorbed by the voltage leads, the internal voltmeter used has a very high impedance.

**Figure 3:**
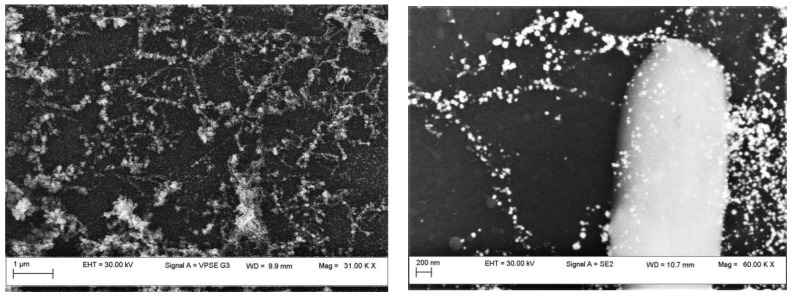
SEM image of a section of the sensor structure (mag: left: 31 and right: 60 KX).

Scanning electron microscopy (SEM) image, as shown in Figure 3, reveals the alignment, between the electrodes, of the carbon nanotubes with Pt nanoparticles deposited on them. The role of the Pt is to facilitate the adherence of the SWCNTs between gold/copper and between individual SWCNTs, knowing that the length of nanotubes is much shorter than the distance between electrodes. Strong adherence is expected to be obtained, which is formed during the drying process.

### 3.2. Analysis and Interpretation of Results

**Figure 4:**
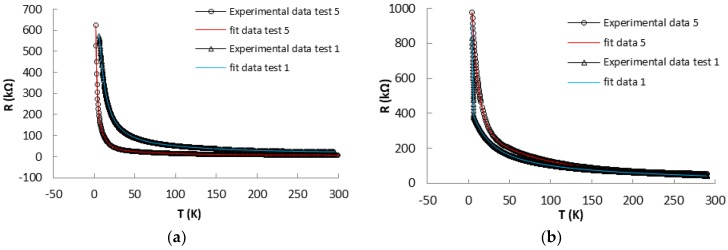
Resistance vs. Temperature (R-T) curves for: Sensor 1 (**a**); and Sensor 2 (**b**).

Both sensors have been cooled several times from room temperature (approximately 300 K) down to approximately 2 K, until resistance reached a constant value with error less than 1%. This procedure can be considered as an ageing process. The resulting variations of resistance with temperature for the studied structures, for two different run tests, initial (Test 1) and after five runs (Test 5), during heating/cooling cycles, are shown in Figure 4. 

The nanotubes based structures showed sensitivity and fast response at temperature variations, having the initial resistance, after the ageing, approximately 7.69 kΩ for Sensor 1 and 55.06 kΩ for Sensor 2. The mechanism of conductivity in SWCNTs structures can be explained by the percolation theory [21,22] and can be calculated as: $\sigma \;=\;\frac{1}{LZ}$, where L is a characteristic length, which depends of the concentration of sites, Z is the resistance of the path with the lowest average resistance. With a decrease in temperature, the charge concentration (electrons and holes) decreases, resulting in a reduced number of charge carriers available for recombination, and, statistically, the conductivity of the CNTs decreases, whereas the resistivity of the CNTs increases [23].

Using a nonlinear fitting algorithm, from the Origin software package, we investigated several mathematical expressions capable of describing the experimental data. For both, Sensor 1 and Sensor 2, the experimental data can be well approximated by the following equation:

(1) R(x) = a0 + a1e−xb1 + a2e−xb2 + a3e−xb3

The value of the coefficients *a_0_, a_1_, a_2_, a_3_, b_1_, b_2_, b_3_* and *R^2^* (Root Mean Square) for Sensor 1 and Sensor 2, corresponding to Test 1 and Test 5, respectively, are listed in Table 1.

**Figure 5:**
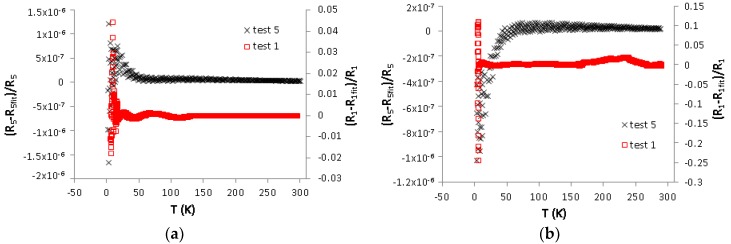
The difference between experimental results and simulated results for: Sensor 1 (**a**); and Sensor 2 (**b**).

The differences between the experimental results and fitted data, Test 1 and Test 5, for Sensor 1 and Sensor 2, are plotted in Figure 5.

In Figure 5, it is shown that the differences between the fitted data and experimental data are very small, suggesting that the empirical equation estimates are almost 100% comparable with the experimental results, especially after undergoing the ageing process ($R^{2}$ = 1).

The R-T characteristics are almost linear in temperature domain from 300 K to 77 K, suggesting an ohmic contact, this being confirmed by [6,7,14,24,25]. Further, our attention was focused on the sensor characterization after ageing process, on the temperature domain below liquid nitrogen temperature (77 K). As the temperature moved down from 77 K to approximately 2 K, the sensor structure resistance increased from 18.3 kΩ to 623 kΩ for Sensor 1, and increased from 153 kΩ to 979 kΩ for Sensor 2, having an allure that suggests a semiconductor behavior (p-type semiconductor) [14,26,27].

**Figure 6:**
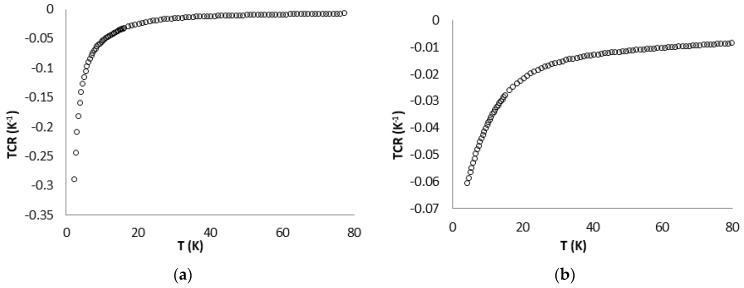
Temperature coefficient of resistance for: Sensor 1 (**a**); and Sensor 2 (**b**).

One of the parameters used to characterize a temperature sensor is the Temperature Coefficient of Resistance (TCR), defined as the relative change of a physical property (resistance) that is associated with a given change in temperature

(2) $$TCR=\frac{R-R_{0}}{R_{0}\left(T-T_{0}\right)}$$

where R is the resistivity of the sensor structure at temperature T and $R_{0}$ is the resistivity at a reference temperature $T_{0}=0\;{}^{\circ}C$ [28]. The plotted results for TCR are presented in Figure 6.

**Figure 7:**
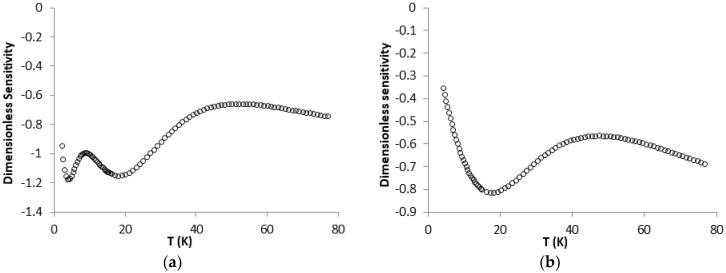
Specific sensitivity for: Sensor 1 (**a**); and Sensor 2 (**b**).

**Figure 8:**
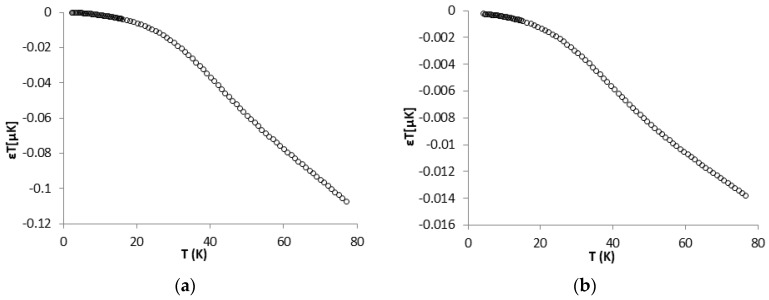
Absolute temperature resolution of: Sensor 1 (**a**); and Sensor 2 (**b**).

In Figure 6, it can be further seen that Sensor 1 has a bigger change in resistance than Sensor 2, especially at temperatures below 20 K, where the TCR average for Sensor 1 is 1.473%/K and for Sensor 2 is 0.365%/K. The CNTs resulted films used in the structures of Sensor 1 and Sensor 2, and the contacts between them together with the terminal electrodes were not affected or destroyed by temperature variations occurring because of possible mismatches at the device interfaces [6]. The sensors, which were tested in the domain 2–300 K with slow heating–cooling cycles, proved to present very good stability. To intensely investigate the R-T curves, we plotted the dimensionless sensitivity (as shown in Figure 7) and absolute temperature resolution (Figure 8) for each structure.

The dimensionless sensitivity SD=(TR)(dRdT) gives the relative temperature sensitivity of the sensor at temperature T. $S_{D}$ ranges from 0.2 to 6 for most common cryogenic temperature sensors, depending on temperature and sensor type [29]. A large specific sensitivity allows the resolution of small temperatures relative to the temperature measured [4], but the temperature range is dependent and, in some situations, is limited by the resistance measurement system accuracy.

The absolute temperature resolution is the smallest temperature change that can be detected, and depends on the sensor characteristics and on the measurement system resolution according to the following expression [4]:

(3) εT = εVI(dRdT)

where $\varepsilon_{T}$ is the temperature resolution of a sensor measuring a temperature T, $\varepsilon_{V}=19.07$ nV is the measurement system resolution, I = 1 mA is excitation current and dRdT is the sensor sensitivity.

From graphics in Figure 7, it is evident that the effect of temperature on the sensor resistance is about the same for both sensor structures. In the domain analyzed here, viz. 2–77 K, Sensor 2 is more sensitive to temperature change than the Sensor 1 due to the nanotube density between the structures’ electrodes and due to the gap dimension between Cu electrodes (Sensor 1) and gold electrodes (Sensor 2).

**Figure 9:**
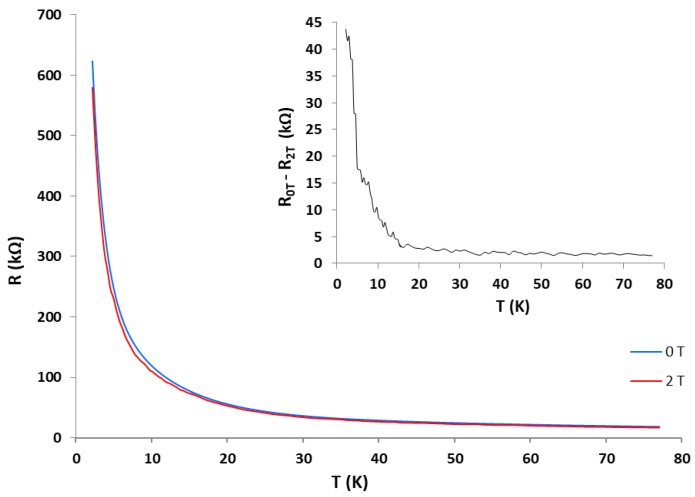
Resistance vs. Temperature curves for Sensor 1 at a magnetic field B = 0 and 2 T. The inset shows the difference of the resistance measured at B = 0 and 2 T.

Additionally, we studied the influence of the magnetic field on the Sensor 1 in the temperature range 2–77 K. Figure 9 shows the temperature dependence of the resistance under the influence of a magnetic field of B = 2 T (Tesla), compared with the characteristic measured at zero field. We conclude that the sensor is sensitive to the magnetic field with steeper drop in resistance, especially at temperatures below 15 K.

## 4. Discussion and Conclusions 

We have investigated resistance vs. temperature characteristic for two different temperature sensors with SWCNTs as sensing element. The sensors were obtained by dropcasting the nanotubes from a solution between two electrodes of a support structure, followed by a dielectrophoresis alignment process and high temperature annealing. Using this methodology, stable layers of aligned SWCNTs were obtained. For temperature range under investigation, i.e., from 300 K to 2 K, we have established an empirical equation for the sensors characteristics R(T) in the form of an exponential equation, consisting of sum of three exponential terms and one constant. This is indicative of a semiconductor behavior for the SWCNTs, and fit the existing data. The differences between experiments and fitted data are very small, thus confirming a very good mathematical approximation of the experimental results. In addition, the structures’ specific sensitivity, thermal coefficient of resistance and absolute temperature resolution are presented. These experimental results show a good reproducibility over the entire temperature range. We highlighted that the SWCNTs based structures were very sensitive, especially in the temperature range 2–77 K. The R(T) characteristic obtained here proves that CNT-based sensors can be successfully used for cryogenic temperature measurements and we expect that it will be intensely studied for temperature measurements for future application.

## Figures and Tables

**Table 1:** The fitting parameters of the Equation (1) corresponding to the experimental data for Sensor 1 and Sensor 2, respectively.

	Sensor 1		Sensor 2	
	Test 1	Test 5	Test 1	Test 5
$a_{0}$	19,222.98068	7.39290915	21,950.90322	48,419.38356
$a_{1}$	88,043.71776	37,752.81212	253,217.90828	142,239.71845
$a_{2}$	254,698.97717	287,769.67141	143,146.18497	142,310.06541
$a_{3}$	801,570.01493	1,555,450	17,913,000	1,092,000
$b_{1}$	94.79632	62.17684	26.24794	77.20495
$b_{2}$	19.37641	7.64496	152.14679	77.21681
$b_{3}$	6.42804	1.49455	1.19707	8.40832
$R^{2}$	0.99997	1	0.99291	1
